# Sleep Paralysis Among Higher Education Students: A Possible Role of Antidepressant and Recreational Stimulant Use

**DOI:** 10.3390/medicina61101844

**Published:** 2025-10-15

**Authors:** Gediminas Gumbis, Kristijonas Puteikis, Rūta Mameniškienė

**Affiliations:** 1 Faculty of Medicine, Vilnius University, LT-03101 Vilnius, Lithuania; 2 Clinic of Neurology and Neurosurgery, Institute of Clinical Medicine, Faculty of Medicine, Vilnius University, LT-03101 Vilnius, Lithuania

**Keywords:** amphetamine, antidepressants, mental health, parasomnias, REM sleep

## Abstract

*Background and Objectives*: While sleep paralysis (SP) is a well-defined disorder, its pathophysiology and causes remain elusive. We aimed to assess the prevalence of sleep paralysis among higher education students and determine factors associated with SP with a focus on psychoactive substance and medication use. *Materials and Methods*: We conducted a cross-sectional online survey across higher education institutions in Lithuania, asking students to report the occurrence and frequency of SP as well as its characteristics and self-rated sleep quality alongside demographic data and history of medication and psychoactive substance use. Subgroup comparisons and correlation analyses were performed in search of factors associated with reported SP. *Results*: The study sample consisted of 275 respondents aged 22.9 ± 4.7 years (240, 87.3% female), 119 (43.3%) of whom reported having experienced SP (average age at first episode 16.4 ± 4.2 years), with 87 (73.1%) more than once. The phenomenology of SP episodes included mostly visual, auditory, sensory, or olfactory hallucinations (73, 61.3%), feelings of fear or anxiety (56, 47.1%), incubus-like phenomena (17, 14.3%), and autonomic symptoms (6, 5.0%). Having experienced SP was associated with the use of antidepressants or recreational stimulant use (χ^2^ = 5.258, *p* = 0.022) as well as higher alcohol intake (Z = −3.568, *p* < 0.001) and lower self-rated sleep quality (Z = −2.413, *p* = 0.016). Earlier age of onset, hallucinations during paralysis, specific time of manifestation during the night, and overall nightmare frequency were related to the recurrence of SP. Respondents tied SP episodes mostly to stress or anxiety (55, 46.2%), the supine sleeping position (31, 26.1%), disturbed sleep cycles (28, 23.5%), and emotional or traumatic experiences (28, 23.5%). *Conclusions*: Our study suggests that SP is prevalent among students with a tendency to recur. We report a correlational association between SP and the use of antidepressants or stimulant drugs, suggesting the need to further explore the possible role of psychoactive agents in this disorder.

## 1. Introduction

Recurrent isolated sleep paralysis (SP) is one of the rapid eye movement (REM)-related parasomnias defined by short (seconds to minutes) episodes of an inability to move the trunk and limbs at sleep onset or upon awakening that are associated with distress and, to fit the definition, should occur in the absence of other sleep disorders or medical conditions [1,2]. While the cause and pathophysiology of SP remain enigmatic, it includes comorbid, environmental, as well as genetic factors [3] and is thought to occur because of a disruption of REM sleep by an arousal response with preserved REM atonia [1]. The prevalence of SP varies across selected groups and has been reported in a systematic review to be 7.6% in the general population, while largely higher among students (28.3%), psychiatric patients (31.9%), and patients with panic disorders (34.6%) [4]. It has also been noted to be higher in ethnic minority groups and females [4]. While the underlying cause of such a variation remains unknown, it may be because of a higher burden of somatic and psychiatric comorbidity, higher rates of substance use, and SP-associated lifestyle factors (such as stress and sleep patterns) in individual groups. Given the relatively high prevalence of SP in selected populations, such as students, the authors suggested wider screening for SP—while this condition is considered to be benign, it may still cause distress; therefore, its identification and explanation for the people affected may help to reduce associated worry while also allowing better insight into the characteristics and pathophysiology of SP.

Experiences during SP have been appraised in folklore, paintings, and the literature across different cultures, but their descriptions and visualisation (e.g., demonic figures in the room or sitting on the sleeping person’s chest) are relatively uniform among people from different backgrounds. A three-factor structural hypothesis in SP has been proposed that includes nightmares of the “intruder” (a sensed presence that may be accompanied by hallucinations, hypothesized to be due to a hypervigilant state of the midbrain), the “incubus” (pressure of the chest and breathing difficulties, thought to occur because of the motoneurons’ hyperpolarization effects on respiration perception), and unusual bodily experiences (e.g., out-of-body experience, attributed to a mismatch between endogenous and exogenous activation of body position systems) [1]. Recently, an overlap between SP and other sleep states, including out-of-body experiences, has been proposed [5], alongside a future focus on the potential therapeutic use of these phenomena.

Student populations, previously shown to be more prone to SP [4], are also among those more significantly affected by psychiatric disorders and substance use, while usually having fewer somatic comorbidities [6]. Sleep quality is an important contributor to well-being in students [7]; thus, a better understanding of sleep disorders and their context in this population may benefit its members. With the recent emergence of reports across different countries examining the prevalence of SP among undergraduates, as well as its relations to local background [8,9,10,11], one of the motivations for the current study was to determine the prevalence and characteristics of SP in Lithuania, the southernmost country of the three Baltic States, whose cultural milieu is shaped by the interplay of paganistic heritage, catholic tradition, and modern Western European influence. To the best of our knowledge, no reports on SP have yet emerged from this region. Moreover, while the association between SP and sleep characteristics, psychological symptomatology, and demographic factors has already been addressed in previous reports [1,2], we sought to supplement this field by exploring the relationship between the occurrence of SP and medication or psychoactive substance use, as well as subjectively perceived provocative factors, setting the stage for future studies on SP prevention and management.

## 2. Materials and Methods

### 2.1. Study Setting

We conducted a cross-sectional anonymous online survey among students across Lithuanian higher education institutions between February 2024 and December 2024. Convenience sampling was used to gather responses by contacting students across all major Lithuanian higher education institutions through closed student social networking groups (used for general or research-related communication, not related to health issues) and mailing lists. Potential participants were invited to “complete a survey on the prevalence of sleep paralysis” with no additional advertisement of the condition. Inclusion criteria were being at least 18 years old, being enrolled in a higher education program, and being able to complete the questionnaire composed in the Lithuanian language. A reported diagnosis of narcolepsy was exclusionary. No additional inclusion or exclusion criteria were used.

### 2.2. Questionnaire

The questionnaire used in the study was created *ad hoc* by the study authors and distributed as a link to a Google Form. A copy of the translated questionnaire is presented in the Supplementary Materials. The questionnaire consisted of items relating to demographic (age, sex, household, and place of residence) characteristics and data on higher education (study type, year, and programme), employment, comorbid conditions, use of psychoactive substances, and medications. Participants were also asked to report on their sleep latency and experiencing nightmares and evaluate their perceived health and quality of sleep on the respective single-item Likert scales ranging from 1 (worst quality) to 10 (best quality). Finally, they were asked about prior experience of SP (an explanation of the condition was provided: “Sleep paralysis episodes are characterised by an inability to move the trunk and limbs when falling asleep and/or waking up. The episodes typically last from a few seconds to a few minutes and may be accompanied by distress, anxiety, and fear of falling asleep”, its age of onset, the frequency of such episodes, as well as associated factors (hallucinations, specific time of occurrence during the night, and medication used). The participants were also encouraged to provide free-text descriptions of hallucinations and other sensations during SP and report the perceived provocative factors of SP episodes. Descriptions of the symptomatology in free text were used, instead of standardised closed-ended questionnaires, to acquire more extensive and more personal accounts of the episodes.

### 2.3. Statistical Analysis

Microsoft Excel v2503 (Microsoft Corporation, Redmond, WA, USA) and IBM SPSS Statistics 26 (IBM Corp., Armonk, NY, USA) were used for statistical analysis. The normality of data distribution was assessed using the Kolmogorov–Smirnov test. Chi-squared or Fisher’s exact tests were used for categorical data, Mann–Whitney U or Kruskal–Wallis tests were applied for subgroup comparisons, and Pearson’s or Spearman’s correlations were used for linear and ordinal data in search of factors associated with having experienced SP, as well as its reported frequency. Ordinal and binary regression analyses were used as a confirmatory method for detecting associations. The threshold for the two-tailed tests was set at *p* < 0.05. The targeted sample size was set to n = 210 as it would allow achieving a power of 1-β = 0.95 and effect size d = 0.5 with α = 0.05 for a two-tailed t-test comparing means within two independent participant subgroups.

### 2.4. Ethics

No ethical approval was required, according to the Lithuanian law of biomedical studies, because of the fully anonymous design of the study (responses that are unidentifiable are not considered biomedical data). Participants provided their informed consent within the online form.

## 3. Results

The study sample consisted of 275 students aged 22.9 ± 4.7 years. The sociodemographic characteristics of the respondents are presented in Table 1, and rates of substance and medication use are shown in Table 2.

**Figure 1:**
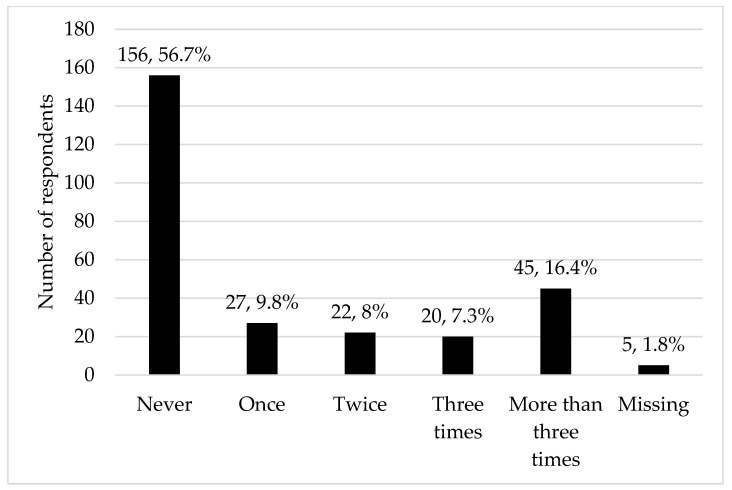
Reported frequency of sleep paralysis episodes during lifetime.

The general sleep and health-related characteristics of the study sample are presented in Table 3. There were 119 (43.3%) respondents who reported having experienced SP (average age at first episode 16.4 ± 4.2 years), most of them more than three times (Figure 1). Among them, 73 (61.3%) reported experiencing hallucinations during the episodes, and 33 (27.7%) noticed specific times of onset during the night. The predominant hallucinations were visual—43 (58.9%) reported seeing shadows, human-like figures or faces (intruders) and 11 (15.1%) saw things, demons or non-human creatures. When reported, the sex of the intruder was predominantly male, except for two respondents reporting seeing female characters from Western horror movies. No characters specific to Lithuanian culture were cited in any of the accounts. Other types of hallucinations included auditory (26, 35.6%), sensory (12.3%), and olfactory (8, 10.9%).

Within the optional free-text response fields of the survey, respondents also explicitly stated having experienced fear or anxiety (56, 47.1%) during SP episodes, some reported incubus-like experiences, chest pressure or difficulty breathing (17, 14.3%), autonomic (tachycardia, diaphoresis, 6, 5.0%) or sensory (pain, feeling hot, 10, 8.4%) symptoms, out-of-body experiences (4, 3.4%), feeling their blanket being pulled from their feet (4, 3.4%), and tinnitus or vertigo (3, 2.5%). The episodes were almost exclusively unpleasant, although one respondent reported feeling good, and some stated becoming acquainted with the episodes and having learnt how to cope with them over time.

Respondents perceived SP occurrences to be associated with stress or anxiety (55, 46.2%), the supine sleeping position (31, 26.1%), disturbed sleep cycles (28, 23.5%), emotional or traumatic experiences (28, 23.5%), and fatigue (24, 20.2%) or insomnia (12, 10.1%). A minority of individuals thought their episodes to be provoked by alcohol use (5, 4.2%), travel to distant destinations (4, 3.4%), or psychoactive substance use (2, 1.7%).

Five (4.2%) respondents sought professional consultations for the episodes, and none perceived them to be related to medication use.

Episodes of SP were reported more often among those who had tried psychoactive stimulants (e.g., cocaine, amphetamines) or were using antidepressants—13 (68.4%) of such respondents (both subgroups coincide in size, their overlap is n = 3) had experienced SP in contrast to 106 (41.4%) individuals experiencing SP with no history of antidepressant or stimulant use (χ^2^ (1, n = 275) = 5.258, *p* = 0.022, φ = 0.138). In a binary regression model, the odds ratio for reported SP among individuals using antidepressants or having tried stimulants was OR = 2.8 (95% confidence interval CI = 1.0 to 7.9, *p* = 0.041) for both variables (the value coincides because the subgroup using antidepressants and the one with history of stimulant use are equivalent in size). Antidepressants used were escitalopram (4, 30.8%), sertraline (3, 23.1%), fluoxetine (2, 15.4%), paroxetine (1, 7.7%), venlafaxine (1, 7.7%), venlafaxine in combination with mirtazapine (1, 7.7%), and unspecified (1, 7.7%). Stimulants reportedly tried by those having experienced SP included 3,4-methylenedioxymethamphetamine (MDMA, used by 8, 61.5%), amphetamine (6, 46.2%), cocaine (5, 38.5%), methamphetamine (1, 7.7%), and unspecified (2, 15.4%). Individuals who had experienced SP were, on average, consuming more alcohol (Z (1, n = 175) = −3.568, *p* < 0.001, r = 0.27) and scored lower on the self-rated sleep quality scale (Z (1, n = 275) = −2.413, *p* = 0.016, r = 0.15).

There was no association between having experienced SP and age, sex, household situation, study characteristics, employment, place of residence, smoking, cannabis or psychedelic substance use, sedative/anxiolytic or sleep medication use, self-rated health, or time to sleep onset (*p* > 0.05).

**Figure 2:**
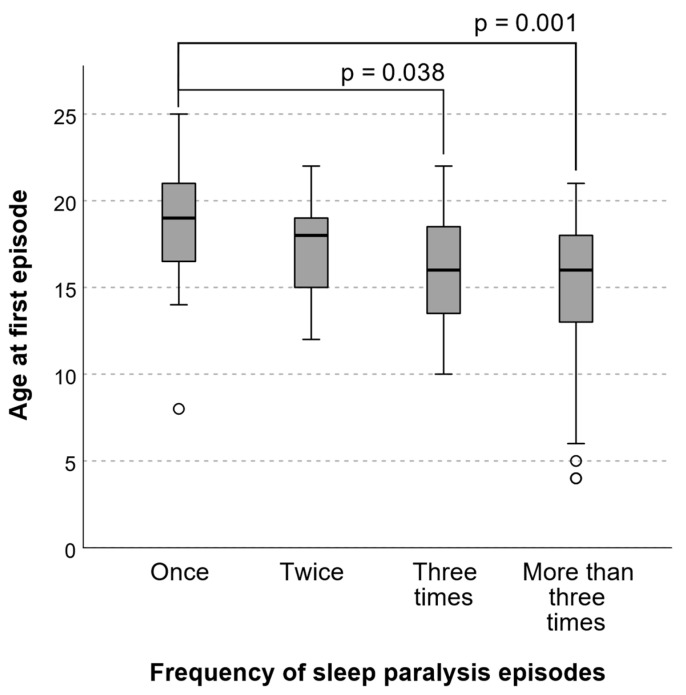
The lifetime prevalence of SP episodes depending on age of onset (Kruskal–Wallis H (3, n = 114) = 18.787, *p* < 0.001, ε^2^ = 0.002). Between-group comparisons are presented after Bonferroni correction. This association remained statistically significant (β = −0.244, *p* < 0.001) after adjusting for age and sex in an ordinal regression model. ○ – data outliers.

The frequency of SP episodes was associated with their onset at an earlier age (Figure 2), reported hallucinations during paralysis (Z (1, 113) = −3.057, *p* = 0.002, r = 0.29), specific time of occurrence during the night (Z (1, 113) = −2.500, *p* = 0.012, r = 0.24), and overall nightmare frequency (n = 114, ρ = 0.340, *p* < 0.001).

## 4. Discussion

We presented the results of a cross-sectional student survey exploring SP and associated factors. The lifetime prevalence of SP was 43.3%, higher than could be expected based on a systematic review in 2011 [4], but similar to more recent reports from Argentina (40.7%), Italy (37.5%), the United States (34.8%), and Poland (31.9%) [8,9,10,11,12]. The phenomenology of SP episodes among Lithuanian students was not exclusive—most consisted of visual or auditory hallucinations alongside negative emotion (fear and anxiety)—and no influences of local cultural context were observed in the content of hallucinations. The rate of visual hallucinations consisting of a being or person compares with another study from the United States [13] that, respectively, reported rates of 25% and 22% for such events (the overall rate of visual anthropomorphic hallucinations in our study was 59%). Similar rates of auditory hallucinations (15–33% vs. 36%) and lower rates of sensory ones (16–58% vs. 12%) were found. The rate of hallucinations by type or content, as well as associated symptoms, varies across studies, but this may be more dependent on survey design than true differences in symptomatology across populations [14]. For instance, in our study, respondents were asked to elaborate on their experience in free text rather than select options within a predefined set of symptoms, possibly leading to a lower prevalence of sensory, autonomic, or emotional symptoms than was reported in a student sample from a culturally similar country [12]. Besides several accounts citing fictional characters from popular culture (horror movies), “demons” or “dragons”, there was no overarching cultural narrative for the content experienced. We believe that this may represent the students’ assimilation to more globalised rather than Lithuanian cultural phenomena, reflecting a diminished role of local storytelling and a loss of affiliation with traditional fiction that no longer prevails in everyday media and entertainment products.

Our results revealed that the occurrence of SP is more frequent with worse ratings of overall sleep quality, a finding reported in earlier studies [10,11,15] that may support the notion of SP reflecting broader sleep dysfunction because of the same predisposing factors (e.g., mental health disorders, disrupted sleep regimen or even genetic phenotype) as well as a bidirectional interaction between SP and other sleep disorders [16]. The reported frequency of SP episodes was related to age of onset (even after adjustment for respondent age) as well as the overall rate of nightmares (the latter result is in line with the one among Czech students [17]) or specific time of SP occurrence, further supporting the view that the presence of SP may overlap with other sleep characteristics [3,16]. Most individuals, however, do not consult a healthcare specialist because of SP alone [10], suggesting that, in clinical settings, it may usually be recognised only alongside more pressing sleep-related problems, such as insomnia.

The association between alcohol consumption and SP in youth is rather well-established [8,18,19] and thought to be linked to the REM-modifying effects of alcohol—while we did not find a higher rate of SP among alcohol users (which comprised two-thirds of the sample), there was a dose-dependent relationship with SP being more likely with heavier alcohol use. We could not confirm the association between smoking and SP, which has been reported previously by some [8,18]. While evidence regarding the role of smoking in SP is mixed [3], our results could have also been influenced by a broader understanding of smoker status (i.e., the inclusion of other nicotine delivery systems than cigarettes), possibly blunting the discrimination between participants used to low versus high nicotine intake. Similarly to a study conducted in Poland [8], a neighbouring country to Lithuania, we report a link between antidepressant use (mostly, selective serotonin reuptake inhibitors, SSRIs) and the occurrence of SP. Two individuals using antidepressants were among the five who had consulted a physician because of SP; thus, antidepressant prescription because of SP cannot be excluded in these two cases. However, the remaining participants who had been prescribed antidepressants may be safely assumed to be using them because of comorbid mental health disorders. While our study provides correlational rather than causative data, it points to the possibility of a rather paradoxical association between SP and the use of antidepressants—a drug class suggested for the treatment of the same disorder [20]—and suggests the need to consider the possible REM-inhibiting effects of antidepressants [21,22,23] that may potentially translate into states within the wake–REM transition, including episodes of SP [5]. Given the mixed results in the literature [3,8], more certainty regarding the link between SP and antidepressants across different subpopulations may emerge with future studies.

We are not aware of reports exploring the impact of psychoactive substance use among individuals reporting SP, beyond alcohol, tobacco, caffeine, and prescription medication [3]. In our study, respondents with prior use of stimulants, such as amphetamines, MDMA, or cocaine, but not those who had used cannabinoids, psychedelics, or anxiolytic medications, were more likely to report the occurrence of SP. The use of stimulating agents, such as modafinil or amphetamines, remains within the array of options for treating excessive daytime sleepiness in narcolepsy—a disorder in which SP is one of the symptoms [24,25]. In our sample, we cannot speculate about the temporal association between SP episodes and the use of stimulants. However, it is known that the latter may have lasting effects on neural circuits [26], potentially allowing for SP to appear even with a delay after use. While the mechanisms of atonia in REM sleep have been established and relate to GABA_B_ and GABA_A_/glycine receptor-mediated inhibition [27], the pathophysiology of hallucinations and associated symptoms remains elusive [28] but is likely to depend on rather wide cortical and limbic activation, suggested to be mediated via 5-HT_2A_ receptors. Therefore, the threshold for transitioning to a state between normal REM sleep and wakefulness, as seen in SP [5], may, in theory, be lowered because of stimulant use-related upregulation of dopaminergic, serotonergic, and noradrenergic networks. However, such an association should be proven by a prospective follow-up of individuals using stimulants. A population of interest for such observational studies could be patients with attention-deficit/hyperactivity disorder who are known to be predisposed to a plethora of sleep disorders and are often prescribed stimulants as the first-line medication [29,30].

Interestingly, only several respondents perceived alcohol or psychoactive substances to be associated with SP, and none thought the latter to be related to the use of prescription medication, in contrast to correlational findings within the study sample. This suggests that the influence of these agents may be relatively subtle and not evident to the individuals affected.

Several limitations of the current study should be acknowledged. Firstly, we used convenience sampling to collect responses, making the study prone to sampling bias, such as a female and social science student predominance, and potentially leading to an overestimation of SP prevalence among higher education students in Lithuania. As individuals with a personal interest in SP may have been more likely to participate, the results are not fully generalizable to the whole student population. This is especially true for male individuals, life sciences, technology, and humanities students who were underrepresented in the current student sample. With the study being set in Lithuania, its findings may also be different from those in other countries. Secondly, the cross-sectional nature of our study prevented us from revealing temporal associations between the potential risk factors for SP and SP episodes, yielding correlational associations with unknown causality and directionality. For instance, the association between antidepressant use and SP may be confounded by an underlying psychiatric comorbidity, with no direct causal interaction. This also applies when considering use of psychoactive substances, especially given the small (n = 3) yet present overlap of individuals reporting both stimulant and antidepressant use—psychiatric conditions, rather than pharmacological factors, may predispose such respondents to SP. While the use of self-reporting during surveying made it possible to assess a wider spectrum of characteristics among the general student population, such data may be less reliable than those emerging from structured in-person interviews or electronic health records. It is also subject to recall bias as participants may forget, misremember, or intentionally misreport past experiences. The use of an *ad hoc* questionnaire, rather than standardised instruments dedicated to SP, while allowing to capture a broader and more nuanced range of experiences in open-ended rather than predefined questionnaire items, may prevent direct comparability with other studies. Finally, the study sample remains limited, leading to such a statistical power that may leave subtle between-group differences unrecognised.

## 5. Conclusions

Our study suggests a relatively high prevalence of SP among higher education students in Lithuania and a tendency for SP to recur in affected individuals. A higher frequency of SP was associated with an earlier onset of the first episode, reported hallucinations, specific time of onset, and the frequency of nightmares. We did not find examples of culturally primed SP episodes, and their characteristics were consistent with the previous literature. Our data indicated that the occurrence of SP is more frequent among students using antidepressants or having used stimulant drugs. While the current study cannot determine any causal relationship between the use of these agents and the occurrence of SP, there is a need for future studies to assess the implications of both prescription medication and recreational substances in the pathophysiology and manifestation of SP.

## Figures and Tables

**Table 1:** Sociodemographic characteristics of the study sample.

Characteristic	n, % or Mean, SD
**Age, years**	22.9 ± 4.7
**Sex**	
Male	29, 10.5%
Female	240, 87.3%
Other	6, 2.2%
**Household situation**	
Lives alone	56, 20.4%
Lives with a partner	79, 28.7%
Lives with parents	80, 29.1%
Lives with family	8, 2.9%
Lives with friends	38, 13.8%
Other	14, 5.1%
**Study type**	
Undergraduate	170, 61.8%
Masters	58, 21.1%
PhD	1, 0.4%
Continuous studies (medicine, law)	46, 16.7%
**Study year**	
1	50, 18.2%
2	110, 40%
3	54, 19.6%
4	51, 18.5%
5	9, 3.3%
6	1, 0.4%
**Study program**	
Life sciences	8, 2.9%
Technology sciences	6, 2.2%
Medicine and health sciences	85, 30.9%
Social sciences	150, 54.5%
Humanitarian sciences	26, 9.5%
**Employment during studies**	
Yes	151, 54.9%
No	124, 45.1%
**Place of residence**	
Village	11, 4.0%
Town	10, 3.6%
City	60, 21.8%
Large city	194, 70.5%

SD—standard deviation.

**Table 2:** Rates of psychoactive substance and medication use in the study sample.

Characteristic	n, % or Mean, SD
**Current smoker status (cigarettes, electronic cigarettes, heated tobacco, pipe, hookah)**	
Yes	110, 40.0%
No	165, 60.0%
**Wakes up to smoke at night**	
Yes	12, 10.9%
No	98, 89.1%
**Current use of any amount of alcohol**	
Yes	182, 66.2%
No	93, 33.8%
**Mean weekly standard units of alcohol consumed**	2.2 ± 2.3
**Any psychoactive substance use**	
Never	175, 63.6%
Yes, during lifetime	63, 22.9%
Yes, in the past year	27, 9.8%
Yes, in the past month	10, 3.6%
**Substances used (use in lifetime, reported by respondents in free text):**	
Cannabis and synthetic cannabinoids	78, 78.0%
Hallucinogens (e.g., LSD, DMT, psychedelic mushrooms)	17, 17.0%
Stimulants (e.g., cocaine, amphetamine, methamphetamine)	19, 19.0%
**Current use of sleep medication**	
No	246, 89.5%
Yes	29, 10.5%
**Sleep medication use frequency**	
No medication	246, 89.5%
Less than several times per month	10, 3.6%
Several times per month	9, 3.3%
Several times per week	3, 1.1%
Every day	7, 2.5%
**Current use of sedatives/anxiolytics**	
No	232, 84.4%
Yes	43, 15.6%
**Sedative/anxiolytic use frequency**	
No medication	232, 84.4%
Less than several times per month	17, 6.2%
Several times per month	12, 4.4%
Several times per week	6, 2.2%
Every day	6, 2.2%
Missing	2, 0.7%
**Current use of antidepressants**	
No	256, 93.1%
Yes	19, 6.9%
**Antidepressant use frequency**	
No medication	256, 93.1%
Less than several times per month	0, 0
Several times per month	0, 0
Several times per week	0, 0
Every day	19, 6.9%

SD—standard deviation.

**Table 3:** General health and sleep characteristics of the study sample.

Characteristic	n, % or Mean, SD
**Any current comorbid disorder**	
No	179, 65.1%
Yes	61, 22.2%
Unknown/cannot say	35, 12.7%
**Any current sleep disorder**	
No	254, 92.4%
Yes	8, 2.9% (5 insomnia, 1 periodic limb movement disorder, 2 unspecified)
Unknown/cannot say	13, 4.7%
**Usual nightmare frequency**	
Never	19, 6.9%
Less than twice a year	47, 17.1%
About twice a year	94, 34.2%
Around twice per month	88, 32.0%
Around twice per week	24, 8.7%
Every night	3, 1.1%
**Usual time to sleep onset after going to bed**	
<5 min	9, 3.3%
5 to 10 min	50, 18.2%
10 to 20 min	80, 29.1%
20 to 30 min	53, 19.3%
30 to 60 min	62, 22.5%
>60 min	16, 5.8%
Missing	5, 1.8%
**Self-rated health**	7.6 ± 1.4
**Self-rated quality of sleep**	7.2 ± 1.7

SD—standard deviation.

## Data Availability

The data of the study are available upon reasonable request accompanied by a study plan.

## Supplementary Materials

The following supporting information can be downloaded at: https://www.mdpi.com/article/10.3390/medicina61101844/s1, Supplementary Table. A translated version of the questionnaire used in the study.
