# Supplementary material for: Sleep Paralysis Among Higher Education Students: A Possible Role of Antidepressant and Recreational Stimulant Use
Source: Medicina (Kaunas). 2025 Oct 15;61(10):1844. doi: 10.3390/medicina61101844 (PMC12566577; doi:10.3390/medicina61101844)
Supplement: Supplementary file 1 [file medicina-61-01844-s001.zip › medicina-3900025-supplementary.pdf]

Supplementary Materials

Supplementary Table. A translated version of the questionnaire used in the study.

What is your age: ______

What is your sex?

- o Male
- o Female
- o Other

What is your household situation?

- o I live alone
- o I live with a partner
- o I live with parents
- o I live with family
- o I live with friends
- o Other: ___________

Which type of studies best apply in your case?

- o Undergraduate
- o Masters
- o PhD
- o Continuous studies (e.g., medicine, law)

What study year are you currently in?

- o 1
- o 2
- o 3
- o 4
- o 5
- o 6

What is your study program?

- o Life sciences
- o Technology sciences
- o Medicine and health sciences
- o Agricultural sciences
- o Social sciences
- o Humanitarian sciences

Are you employed while studying?

- o Yes
- o No

What is your place of residence?

- o Village
- o Town
- o City
- o Large city

Are you currently a smoker (including cigarettes, electronic cigarettes, heated tobacco, pipe or hookah)?

- o Yes
- o No

If yes, do you wake up to smoke at night?

- o Yes
- o No

Have you currently been using any amount of alcohol?

- o Yes
- o No

If yes, please indicate the usual number of standard units of alcohol (one unit is a half a pint of average-strength beer, a glass of wine or a shot of spirit) consumed per week: _____________

Have you ever used psychoactive substances?

- o Never
- o Yes, during lifetime
- o Yes, in the past year
- o Yes, in the past month
- o If yes, please indicate the substance (or substances): _________

Do you have any medical conditions?

- o No
- o Yes
- o Unknown/cannot say

If yes, please indicate: __________

How would you rate your overall health on a scale from 1 to 10 (1 – worst health possible, 10 – best health possible)

○1 ○2 ○3 ○4 ○5 ○6 ○7 ○8 ○9 ○10

How would you rate your overall sleep quality on a scale from 1 to 10 (1 – worst sleep quality possible, 10 – best sleep quality possible)

○1 ○2 ○3 ○4 ○5 ○6 ○7 ○8 ○9 ○10

Have you been diagnosed with any sleep disorders?

- o No
- o Yes

Unknown/cannot say

If yes, please indicate:

- o Insomnia
- o Circadian rhythm sleep disorder
- o Periodic limb movements
- o Obstructive sleep apnoea
- o Hypersomnia
- o Narcolepsy
- o Restless legs syndrome
- o Other: _____________

How often do you have nightmares?

- o Never
- o Less than twice a year
- o About twice a year
- o Around twice per month
- o Around twice per week
- o Every night

Do you currently use any sleep medication?

- o No
- o Yes

If yes, indicate the frequency of sleep medication use

- o No medication
- o Less than several times per month
- o Several times per month
- o Several times per week
- o Every day

Do you currently use any sedatives/anxiolytics?

- o No
- o Yes

If yes, indicate the frequency of sedative/anxiolytic use

- o No medication
- o Less than several times per month
- o Several times per month
- o Several times per week
- o Every day

Do you currently use any antidepressants?

- No
- Yes

If yes, indicate the frequency of antidepressant use

- No medication
- Less than several times per month
- Several times per month
- Several times per week
- Every day

How much time does it usually take for you to start sleeping after going to bed?

- <5 minutes
- [5 to 10) minutes
- [10 to 20) minutes
- [20 to 30) minutes
- [30 to 60] minutes
- >60 minutes

The following items relate to sleep paralysis. Sleep paralysis episodes are characterised by an inability to move the trunk and limbs when falling asleep and/or waking up. The episodes typically last from a few seconds to a few minutes and may be accompanied by distress, anxiety, and fear of falling asleep.

How many times did you experience sleep paralysis?

- Once
- Twice
- Three times
- More than three times
- Never

If you have experienced sleep paralysis, how old were you during the first episode? _______________

If you have experienced sleep paralysis, did you experience hallucinations during these episodes?

- Yes
- No

If yes, please describe them: _____________

Please also describe any other sensations during the episodes: _________

Do you feel that there are specific factors provoking these episodes?

- Insomnia
- Disturbed sleep cycles
- Stress or anxiety
- Emotional or traumatic experiences
- Travel to distant destinations
- Alcohol use
- Psychoactive substance use
- Supine sleeping position
- Fatigue
- Other: __________

Do you feel that these episodes occur at a specific time?

- Before sleep onset
- Upon awakening at nigh
- Upon awakening in the morning
- Other: __________

Do you feel that the medication you use is associated with these episodes?

- Yes
- No

Did you seek professional consultations for the episodes?

- Yes
- No
